# Transcranial direct current stimulation (tDCS) over vmPFC modulates interactions between reward and emotion in delay discounting

**DOI:** 10.1038/s41598-019-55157-z

**Published:** 2019-12-10

**Authors:** Aurélie L. Manuel, Nicholas W. G. Murray, Olivier Piguet

**Affiliations:** 1 The University of Sydney, School of Psychology, Sydney, Australia; 2 The University of Sydney, Brain & Mind Centre, Sydney, Australia; 3 ARC Centre of Excellence in Cognition & its Disorders, Sydney, Australia; 4 Macquarie University, School of Psychology, Sydney, Australia

**Keywords:** Cognitive control, Prefrontal cortex

## Abstract

Delay discounting requires computing trade-offs between immediate-small rewards and later-larger rewards. Negative and positive emotions shift decisions towards more or less impulsive responses, respectively. Models have conceptualized this trade-off by describing an interplay between “emotional” and “rational” processes, with the former involved during immediate choices and relying on the ventromedial prefrontal cortex (vmPFC), and the latter involved in long-term choices and relying on the dorsolateral prefrontal cortex (dlPFC). Whether stimulation of the vmPFC modulates emotion-induced delay discounting remains unclear. We applied tDCS over the vmPFC in 20 healthy individuals during a delay discounting task following an emotional (positive, negative) or neutral induction. Our results showed that cathodal tDCS increased impulsivity after positive emotions in high impulsivity trials. For low impulsivity trials, anodal tDCS decreased impulsivity following neutral induction compared with emotional induction. Our findings demonstrate that the vmPFC integrates reward and emotion most prominently in situations of increased impulsivity, whereas when higher cognitive control is required the vmPFC appears to be less engaged, possibly due to recruitment of the dlPFC. Understanding how stimulation and emotion influence decision-making at the behavioural and neural levels holds promise to develop interventions to reduce impulsivity.

## Introduction

Would you like to receive $50 now or $70 in 40 days? Decisions of this nature are prevalent in everyday life, for example when electing to save for our children’s education, our retirement or investing money in the stock market^1^. These decisions are typically investigated using the delay discounting task and require computing a trade-off between time and gains/losses^2^. Understanding how people make choices on experimental delay discounting tasks is of high importance as performance on these tasks correlates with real-word behaviours. For example, choosing larger-later rewards over smaller-sooner rewards is associated with better academic performance, social relationships and more adaptive social functioning^3,4^. Conversely, choosing smaller-sooner over larger-later rewards is associated with a wide range of impulsivity-related pathological conditions, including drug dependence^5^, gambling^6^ or food-related disorders^7,8^.

Although individuals’ delay discounting rate is thought to be fairly stable and even heritable^9^, decisions can be shifted towards either more patient or more impulsive choices^10^. Emotions for instance play a key role in modulating decision-making^11^. Positive emotions tend to shift decisions towards larger-later rewards on the delay-discounting task, whereas negative emotions have the opposite effect with an increase in impulsive choices^12–15^, although the reverse has also been shown^16,17^.

The fact that emotion processing and decision-making interact is not surprising, given the underlying and overlapping brain networks^18,19^. Delay discounting tasks consistently involve a reward network comprising the ventromedial prefrontal cortex (vmPFC) and the striatum in fMRI studies^20–23^. Similarly, lesion^24,25^ or atrophy^26^ of vmPFC increases the preference for smaller-sooner over larger-later rewards compared with healthy controls or control lesions. Additionally, the vmPFC has extensive connections to the amygdala^27^, a key region for processing emotions and rewards^28^. Amygdala damage or disconnection between vmPFC and the amygdala impairs performance on delay discounting tasks in rodents^29–31^ and contributes to deficits on different decision-making tasks in humans^32–37^. The amygdala is also sensitive to the magnitude effect - a well-known effect described as  greater discounting of low-magnitude compared to high-magnitude rewards^2,38,39^ - and shows greater activity when immediate rewards are preferred^40,41^.

How choices are made in the delay discounting task remains a longstanding and ongoing debate. According to existing models of decision-making, the vmPFC and its connections to the amygdala and striatum are part of the core valuation system^1,20,22,42,43^ preferentially engaged for immediate rewards (also referred to as the “hot” system). This system is also preferentially influenced by physiological modifications, such as those sensed during emotion processing^44^. The dlPFC and anterior cingulate cortex (ACC) are engaged when increased cognitive control is required and more patient decisions are taken (i.e. the “cold” system)^1,45^.

Here, we present a single-blinded crossover placebo-controlled study to determine how vmPFC stimulation affects decision-making and the interactions between emotions and decision-making. To achieve this goal, we designed an emotional delay discounting task where each choice is either preceded by a positive, negative or neutral emotional picture. We targeted the vmPFC using tDCS, a well-tolerated brain stimulation technique where low intensity electrical current is applied to the scalp, to temporarily modulate the underlying cortical excitability^46^. Two stimulation types were used, namely anodal stimulation which increases cortical excitability and cathodal polarization which decreases it^46–48^. Based on the assumption that the vmPFC, and its connections to the amygdala, is a key node of the “hot” decision-making network involved in delay discounting, we anticipated that active tDCS over the vmPFC would modulate delay discounting rate in emotional conditions, and more so in conditions where immediate choices are preferred (i.e. low-magnitude trials).

So far, tDCS studies using delay discounting tasks have targeted the dlPFC^49–55^ or dlPFC and vmPFC simultaneously^45^, but not the vmPFC alone. Resolving this issue has implications for understanding how emotion and decision-making interact at the behavioural and neural levels and whether tDCS has the potential to be used as an intervention to reduce impulsivity.

## Methods

### Participants

Twenty healthy participants aged 24 ± 5 years (19–38 years; 6 males) gave written informed consent to take part in the study and were remunerated for their participation. No participant had a history of neurological or psychiatric illness. All procedures were approved by the University of Sydney Ethics Committee and were performed according to the Declaration of Helsinki.

### Experimental design

Each participant completed three sessions, differing by stimulation condition (anodal, cathodal and sham), separated by a one-week washout period between stimulation conditions. Each tDCS session consisted of three blocks of a delay-discounting task composed each of either positive, negative and neutral emotional pictures. Both stimulation conditions and blocks were randomised for each individual.

### Delay discounting task

The ability to delay gratification was assessed with Monetary Choice Questionnaire (MCQ)^56^. The MCQ comprises 27 dichotomous choices and requires participants to choose between a small, immediate monetary reward or a larger, delayed monetary reward (e.g. “Would you prefer $15 today or $35 in 13 days?”). Estimates of delay discounting were calculated for different reward magnitudes, categorised as low-magnitude ($25–35), medium-magnitude ($50–60), and large-magnitude ($75–85). Indifference points were inferred from the choice data and then *k*, a free parameter, that best fit these indifference points was selected, assuming a hyperbolic function. The delay discounting rate was calculated using the following hyperbolic discounting equation: V = A/(1 + *k*D)^57^, where V represents the present value of the delayed reward A at delay D. Larger values for *k* indicate a preference for smaller immediate rewards, and indicate a reduced ability to delay gratification, i.e. increased impulsivity. To account for skewness, *k* values were log-transformed. This procedure is a common practice in the delay discounting literature due to non-normal distributions^58^.

**Figure 1:**
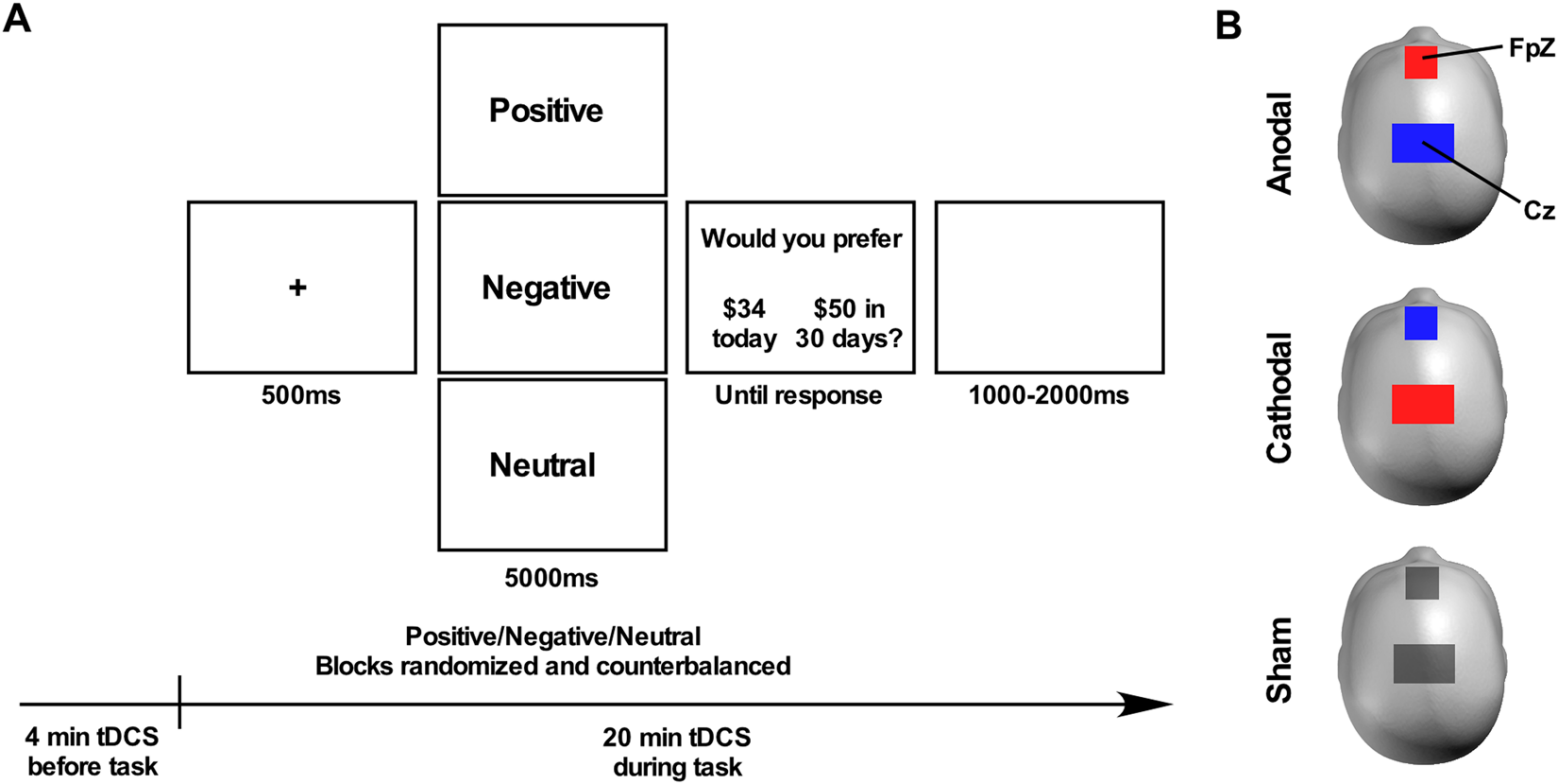
(**A**) Experimental design. The delay discounting task consisted of three blocks containing either positive, negative or neutral pictures presented in randomized order. Participants were first instructed to vividly imagine witnessing the picture and then asked to make a choice on the delay discounting task. (**B**) tDCS montage. Placement of the transcranial direct current stimulation (tDCS) on Fpz (corresponding to the ventromedial prefrontal cortex) and Cz. Red = anodal tDCS; blue = cathodal tDCS; grey = sham tDCS. For sham stimulation, the position of the anodal and cathodal electrodes alternated between participants and stimulation was turned off after 30 seconds.

Before each choice, participants were shown a picture (positive, negative or neutral) and were asked to vividly imagine that they were witnessing the event/content depicted in it (Fig. 1A). Each trial began with a fixation cross presented on a 21.5-inch monitor for 500 ms, followed by a picture (positive, negative or neutral) displayed for 5000 ms. Then, a screen containing both choices was displayed until participants responded. A white screen preceded the next fixation cross and was presented for a random inter-trial interval of 1000–2000 ms. Participants had to indicate their choices by pressing the left or right arrow of a keyboard, according to the choice displayed on left or right of the screen. Each block lasted five minutes and included images of a single valence category (positive, negative or neutral) with a break of two minutes between blocks to reduce any carryover effects of emotion. This mode of presentation was used to distinguish the modulating effects of emotion on decision making and has been used previously in decision making research^59^. Blocks were counterbalanced across individuals and sessions, therefore minimising the risk of fatigue or reduced attention on decision making. Stimulus delivery and subjects’ responses were controlled using E-prime 2.0 software (Psychology Software Tools, Pennsylvania, USA).

The pictures (n = 81) were high-quality positive, negative and neutral photographs chosen from the Nencki Affective Picture System (NAPS)^60^. Pictures were categorised based on their original valence rating^60^ (scale from 1–9; 1 = very negative, 5 = neutral, 9 = very positive) as positive (mean ± SD = 7.9 ± 0.2), negative (2.5 ± 0.3) or neutral (5.1 ± 0.2; repeated measure ANOVA: F_(2,80)_ = 2628.93, *p* < 0.01). Arousal ratings also differed between positive (4.1 ± 0.1), negative (6.7 ± 0.5) and neutral pictures (4.8 ± 0.4; F_(2,80)_ = 105.97, *p* < 0.01). Stimuli were matched with respect to their luminance (F_(2,80)_ = 0.63, *p* = 0.53), contrast (F_(2,80)_ = 2.01, *p* = 0.14) and entropy (F_(2,80)_ = 2.02, *p* = 0.14).

### Transcranial direct current stimulation

Direct electrical current was delivered by a battery-driven stimulator (HDCStim, Newronika s.r.l, Milano, Italy) using two electrodes enclosed in saline soaked sponges (Fig. 1B). The active 5 × 5 cm electrode (which determined whether the stimulation was termed anodal or cathodal) was positioned over Fpz of the 10–20 EEG system to modulate the vmPFC. The reference 6 × 8.5 cm electrode was placed horizontally over the vertex (Cz of the 10–20 EEG system). We used electrodes of two different sizes as it has been suggested to decrease brain current density and functional efficacy under the reference electrode^61,62^.

A typical and safe stimulation protocol of 2 mA with a 30 ms ramp-in and ramp-out period was delivered^63–65^. The stimulation began 4 minutes prior to the delay discounting task and lasted for the remaining 20 minutes of the experiment for active conditions. For the sham condition, the current was turned off after 30 seconds. All subjects were blinded to the tDCS intervention. The impedance was kept below <10 Ω for all stimulation sessions.

### Modelling of tDCS stimulation

**Figure 2:**
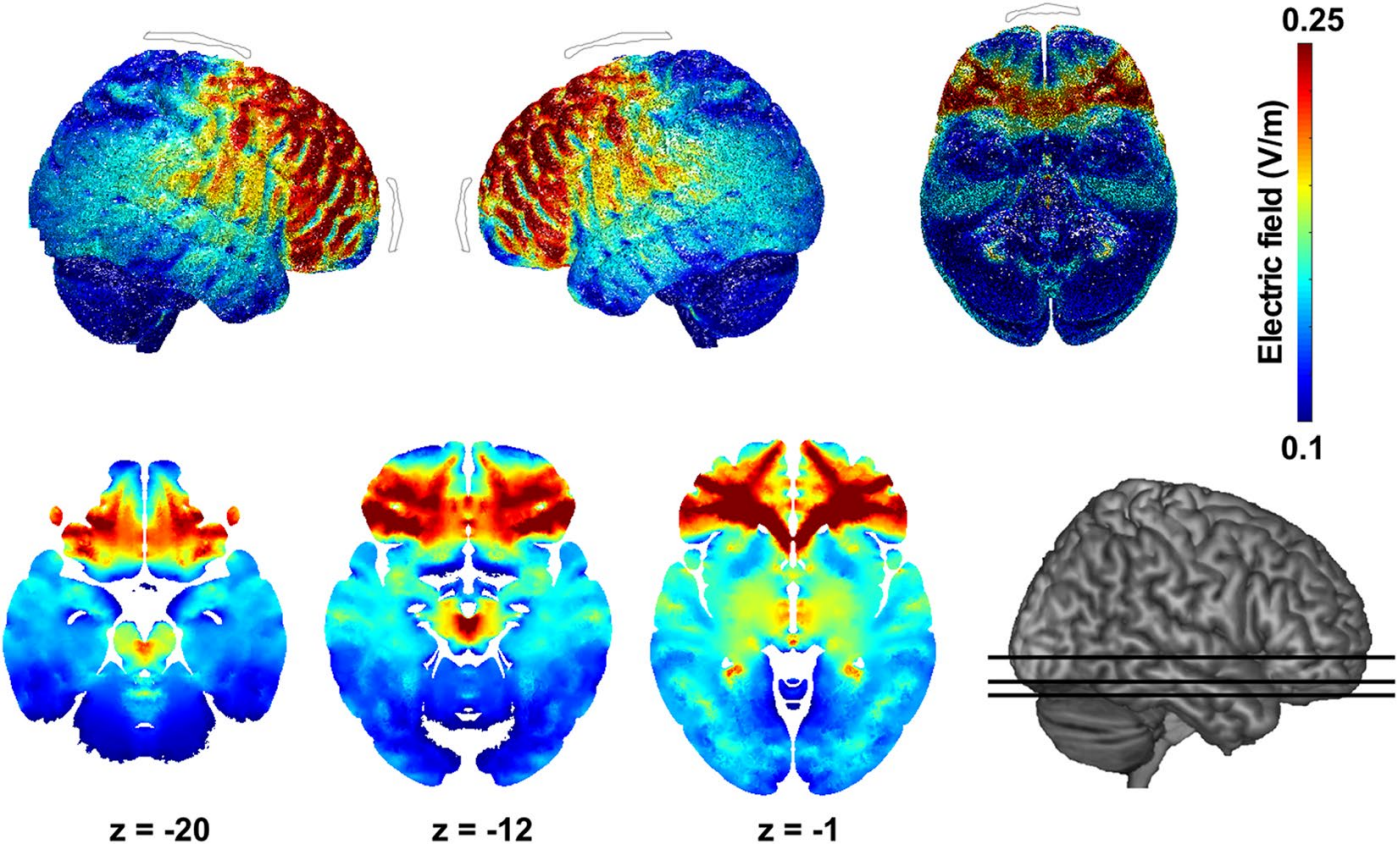
Modelling of tDCS-induced electrical fields. The predicted magnitude of induced electrical field following tDCS viewed from right, left and bottom of the brain as well as displayed on axial slices. Red indicates areas of maximal current density. Grey patches indicate position of electrodes.

Electrical fields induced by tDCS were modelled to assess the areas of underlying brain modulation (Fig. 2). We used the New York Head, the standard head model within ROAST (Realistic, vOlumetric Approach to Simulate Transcranial electric stimulation), to predict the electrical field distribution using our specific stimulation parameters (i.e., electrode placement, size and injected current)^66–68^. The electric field values for anodal stimulation were scaled for 2 mA of inward current with a 5 × 5 cm pad electrode placed on FpZ (2 mA) and a 6 × 8.5 cm pad electrode placed on Cz (−2mA). For cathodal stimulation, a 5 × 5 cm pad electrode was placed on Fpz (−2mA) and a 6 × 8.5 cm pad electrode on Cz (2 mA). This methodology has been utilised in the past where individual MRIs are not available^69–71^. The objective of the model was to predict whether significant current reached our region of interest for this study, i.e., the vmPFC. The model was based on a high-resolution magnetic resonance imaging of an adult male and segmented for six tissue types (scalp, skull, cerebrospinal fluid, gray matter, white matter, air cavities) at 0.5 mm^3^ resolution^72^.

The tDCS current-flow for our electrode montage predicted that stimulating with an active electrode over the Fpz induced wide-spread frontal modulation. Importantly, it confirmed that this montage achieved a significant current flow in the vmPFC. This finding is in line with previous studies that have used similar montages^73^.

### Questionnaires

Participants completed a number of baseline questionnaires assessing impulsivity (Barratt Impulsivity Scale-11, BIS-11^74^), delayed gratification (Delaying Gratification Inventory, DGI^75^, time orientation (Zimbardo Time Perspective Inventory, ZTPI^76^ and ability to flexibly plan and maintain goal-directed behaviours (Self-Regulation Questionnaire, SRQ^77^. Moreover, at the end of the whole experiment, participants rated valence and arousal for a subset of pictures (n = 15) of each emotion category on a scale from 1 to 9 (valence: 1 = very negative, 9 = very positive; arousal: 1 = relaxed to 9 = aroused).

### Statistical analyses

An automated scoring spreadsheet developed by Kaplan^78^ was used to calculate the average *k* (Average *k*(log)) and an individual *k* for low-, medium- and high-magnitude rewards (*k*(log)).

We investigated Average *k*(log) with a 3 × 3 repeated measures ANOVA (rmANOVA) with within factors tDCS (Anodal, Cathodal, Sham) and Emotion (Positive, Negative, Neutral). We also investigated the magnitude effect using a 3 × 3 × 3 rmANOVA with factor Reward Magnitude (Low, Medium, High), tDCS (Anodal, Cathodal, Sham) and Emotion (Positive, Negative, Neutral). Significant interactions were followed by simple effects at each combination of levels of the other factors, and paired t-tests when appropriate, thus reducing the number of post-hoc comparisons. Pearson correlations between questionnaires and performance of the delay discounting task (Average *k*(log)) were analysed to determine potential trait influence on participants’ responses. Data were analysed using IBM SPSS Statistics, 24.0 (SPSS Inc., Chicago, Ill., USA). Effect sizes are reported using the partial eta-square (η^2^).

## Results

### Questionnaires

As anticipated, pictures were judged as positive, negative and neutral by participants. Valence ratings (mean ± SD) differed significantly between positive (7.3 ± 1.0), negative (2.5 ± 0.8) and neutral pictures (4.9 ± 0.6; F_(2,38)_ = 146.876, *p* < 0.001). Arousal ratings also differed between positive (2.6 ± 1.2), negative (6.6 ± 1.0) and neutral pictures (4.6 ± 1.3; F_(2,38)_ = 95.796, *p* < 0.001).

None of the baseline questionnaires (BIS-11, DGI, ZTPI and SRQ) significantly correlated with Average *k*(log) (all *p* > 0.135).

### Delay Discounting Task

**Figure 3:**
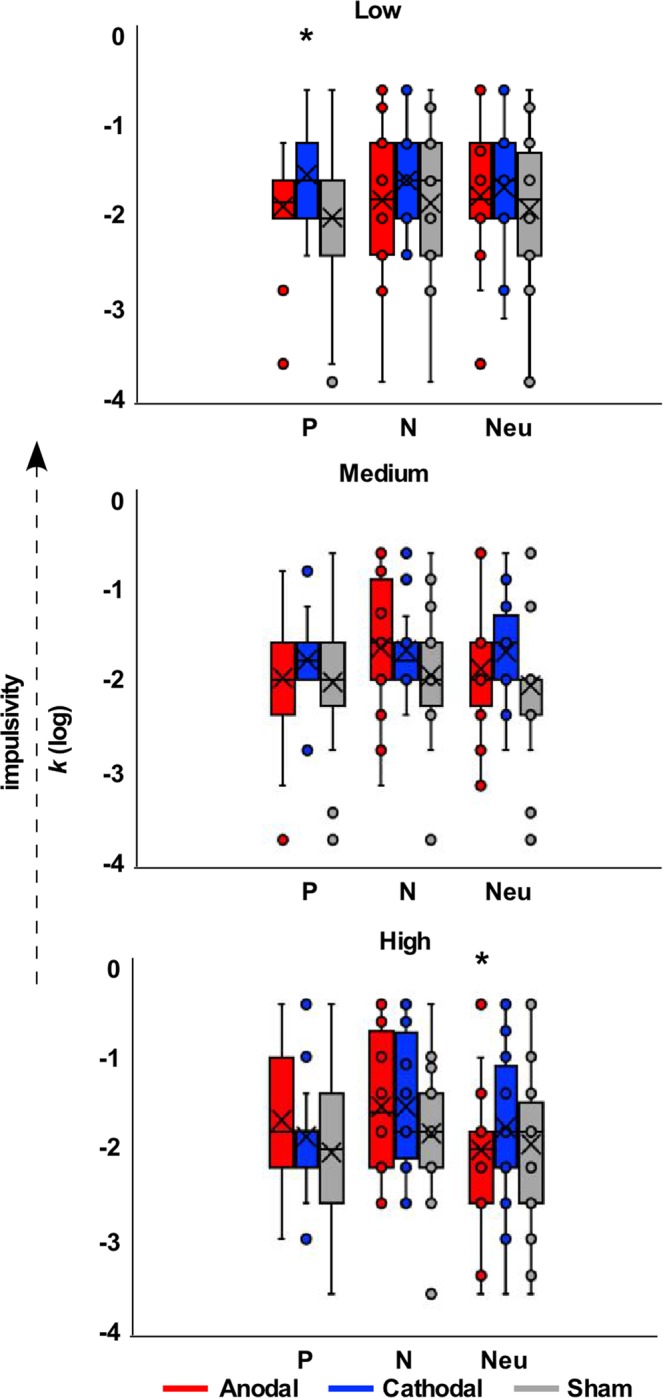
Behavioural results. Discount rates (k, log transformed) for each reward magnitude (Low, Medium, High), tDCS stimulation condition (Anodal, Cathodal, Sham) and Emotion (Positive, Negative, Neutral). *Indicates significant post-hoc differences (p < 0.05).

The 3 × 3 rmANOVA on Average *k* (log*k*) did not reveal any significant main effect of tDCS (F_(2,38)_ = 2.450, *p* = 0.100, η_p_^2^ = 0.114), Emotion (F_(2,38)_ = 1.647, *p* = 0.206, η_p_^2^ = 0.080), or interaction between tDCS and Emotion (F_(4,76)_ = 0.492, *p* = 0.741, η_p_^2^ = 0.025). The 3 × 3 × 3 (Reward Magnitude, tDCS, Emotion) rmANOVA showed a main effect of Reward Magnitude (F_(2,38)_ = 6.763, *p* = 0.003, η_p_^2^ = 0.263), with low- (*p* = 0.005) and medium-magnitude (*p* = 0.012) rewards leading to increased delay discounting compared to larger-magnitude rewards (i.e. the magnitude effect) (Fig. 3). Importantly, there was a significant Reward Magnitude x tDCS x Emotion interaction (F_(8,152)_ = 2.417, *p* = 0.017, η_p_^2^ = 0.113). Simple effect analyses revealed that the interaction was driven by specific effects for low- and high-magnitude rewards. For low-magnitude rewards, a significant difference was present between tDCS conditions when positive pictures were presented (F_(2,38)_ = 3.916, *p* = 0.028, η_p_^2^ = 0.171), such that cathodal stimulation led to significantly more delay discounting compared to both sham (*p* = 0.036) and anodal (*p* = 0.043) conditions. For high-magnitude rewards, a significant difference was observed between emotion conditions for anodal stimulation (F_(2,38)_ = 5.254, *p* = 0.010, η_p_^2^ = 0.217), such that neutral pictures led to decreased delay discounting compared to both positive (*p* = 0.022) and negative (*p* = 0.022) pictures.

## Discussion

This study was designed to determine the role of emotion on individuals’ delay discounting and whether stimulation of the vmPFC shifted choices on this task. Our findings reveal that tDCS over the vmPFC significantly affected delay discounting for specific emotions and reward magnitudes. In conditions of increased impulsivity (i.e. low-magnitude rewards), cathodal stimulation of the vmPFC shifted decisions towards more impulsive choices following positive pictures. In conditions where impulsivity level is lower (i.e. high-magnitude rewards), anodal stimulation decreased delay discounting in non-emotional conditions.

This first finding is in line with our hypothesis and indicates interactions between emotion and decision-making at the behavioural and neural levels. While we did not find evidence for a modulation of delay discounting rate when all trials where combined, we did, as expected, find a modulation when participants were the most impulsive. Stimulation of the vmPFC modulated emotional delay discounting in conditions of high impulsivity. This finding supports models suggesting that the vmPFC is the key node involved in “hot” decision-making processes and preferentially influenced by physiological modifications, such as those sensed during emotion processing^44^. Facing an emotional and impulsive trial, stimulation of the vmPFC may have modulated activity in the amygdala, a structure known for exhibiting greater activity when immediate rewards are preferred^40,41^ and when processing emotions^79–81^. Delay discounting in the emotional trials may have therefore relied more heavily on limbic structures such as the amygdala therefore hampering the effect of vmPFC stimulation on average delay discounting. Alternatively, as low-magnitude rewards require less cognitive demand, they may have been processed more readily in the vmPFC than the dlPFC^82^. Previous literature indeed suggests that in situations where affective salience or cognitive stress is stronger, there is an increased preference for immediate rewards through alterations in dopaminergic signalling and reduced activation of the dlPFC^83–86^. Interestingly, the modulating effect of stimulation was only observed under the positive emotion condition. While somewhat unanticipated, positive cues have been previously shown to lead to increased impulsivity in delay discounting tasks^16,87^ and that the characteristic effects of positive imagery can be dampened with cathodal stimulation over the vmPFC^87–91^. Importantly, this interactive effect was only seen in the context of small rewards. Indeed, small rewards appear to preferentially activate the “hot” system (i.e. the vmPFC), as the decision itself requires less cognitive demand^85^. This preferential activation of the vmPFC, based on reward magnitude, may have resulted in a potential resistance to the inhibitory effect of stimulation; however, an interactive effect of stimulation on emotion is still present, whereby cathodal stimulation reduced the characteristic effect of positive emotion on decision making. The association between the vmPFC and positive emotion is also congruent with neuroimaging studies showing that pleasant cues in particular activate a network comprising the vmPFC and amygdala^87–89^.

Our second finding shows that during anodal stimulation of the vmPFC, individuals were more patient when viewing neutral pictures compared to emotional pictures. Our results are consistent with a study reporting more patient responses in neutral conditions of delay discounting tasks compared to both positive and negative conditions^92^. More patient responses for neutral pictures was also associated with greater vmPFC activity in that same study^92^, suggesting that anodal tDCS increased vmPFC recruitment, in turn leading to a decrease in delay discounting. Another, alternative, explanation is that “cold” processes, supported by the dlPFC, were mostly engaged for the high-reward magnitudes as they require greater cognitive control compared to low-magnitude rewards^1^.

Our model of current flow suggests that tDCS reached the targeted brain region (i.e. the vmPFC). The head model calculated for our tDCS montage and those based on previous studies have shown that this tDCS setup reaches medial frontal areas^73,93^ and increases activation and functional connectivity in the vmPFC, as well as in the amygdala, ACC and striatum^94^. On the basis of the present data alone, one cannot clearly determine to what extent the effect was driven by vmPFC or dlPFC, as our model also predicts intense current flow in the dlPFC. The distribution and directionality of electrical fields during stimulation^95^ as well as the white matter anisotropy conductivity^96–99^ vary greatly across individuals, hence potentially influencing the direction of current flow^95,100–102^. While individual MRIs would enable a more precise mapping of current flow, the use of an MRI-derived standard head model (New York Head) remains a well validated method for estimating the current flow of stimulation in the absence of individual imaging^69–71^. Future studies may incorporate more focal forms of stimulation (e.g., high definition-tDCS, transcranial magnetic stimulation) and apply specific algorithms to determine the optimal montage for targeted stimulation^102,103^. While these approaches are beyond the scope of this study, they will ultimately improve our ability to estimate current flow when aiming for specific regions of interest (i.e. vmPFC/dlPFC). They will also improve our understanding of the individual contributions of these regions towards emotion-induced delay discounting. Testing this paradigm in patients with vmPFC/dlPFC lesion or atrophy, and stimulating the vmPFC/dlPFC (both separately and concurrently) will also clarify the interactions between decision-making and emotion processing in a causal manner.

Understanding the exact conditions under which tDCS modulates delay discounting is of crucial importance to uncover the myriad of negative health behaviours associated with increased delay discounting in clinical populations and propose new interventions. A recent study showed promising findings; stimulation of the dlPFC twice a day for 5 days in a clinically impulsive sample decreased impulsivity on a decision-making task, with effects lasting up to 2 month follow-up^104^. These findings also have clinical implications when considering treatment goals aimed at improving decision-making, for example in patients with vmPFC lesions or in individuals experiencing an inability to regulate emotions and/or with mood disorders^18,26,92,105^. Finally, these results also contribute to the broader understanding of decision-making, in economic decisions for example, where intense emotion can affect financial decisions in complex situations (e.g. stock market)^106^.

## Conclusions

In conclusion, our findings provide support for a role of the vmPFC in emotion-induced decision making, most notably when the immediate rewards are preferred^34,107^. Importantly we demonstrate that the vmPFC drives our decisions and triggers emotional states by shifting decisions based on situational factors, such as cognitive demand and emotional salience^45,85,108,109^. When higher cognitive control is required, and more patient rewards are preferred, the vmPFC appears to be less engaged, due to possible preferential recruitment of “cold” processes mediated by the dlPFC.

## Data Availability

The datasets generated during and analysed during the current study are available from the corresponding author on reasonable request.
